# A versatile transgenic allele for mouse overexpression studies

**DOI:** 10.1007/s00335-015-9602-y

**Published:** 2015-09-14

**Authors:** Hamid Dolatshad, Daniel Biggs, Rebeca Diaz, Nicole Hortin, Christopher Preece, Benjamin Davies

**Affiliations:** Wellcome Trust Centre for Human Genetics, University of Oxford, Roosevelt Drive, Oxford, OX3 7BN UK

## Abstract

For the analysis of gene function in vivo, gene overexpression in the mouse provides an alternative to loss-of-function knock-out approaches and can help reveal phenotypes where compensatory mechanisms are at play. Furthermore, when multiple lines overexpressing a gene-of-interest at varying levels are studied, the consequences of differences in gene dosage can be explored. Despite these advantages, inherent shortcomings in the methodologies used for the generation of gain-of-function transgenic mouse models have limited their application to functional gene analysis, and the necessity for multiple lines comes at a significant animal and financial cost. The targeting of transgenic overexpression constructs at single copy into neutral genomic loci is the preferred method for the generation of such models, which avoids the unpredictable outcomes associated with conventional random integration. However, despite the increased reliability that targeted transgenic methodologies provide, only one expression level results, as defined by the promoter used. Here, we report a new versatile overexpression allele, the promoter-switch allele, which couples PhiC31 integrase-targeted transgenesis with *Flp* recombinase promoter switching and *Cre* recombinase activation. These recombination switches allow the conversion of different overexpression alleles, combining the advantages of transgenic targeting with tunable transgene expression. With this approach, phenotype severity can be correlated with transgene expression in a single mouse model, providing a cost-effective solution amenable to systematic gain-of-function studies.

**Electronic supplementary material:**

The online version of this article (doi:10.1007/s00335-015-9602-y) contains supplementary material, which is available to authorized users.

## Introduction

To explore gene function in vivo, there is a choice to be made between loss-of-function and gain-of-function strategies. Reducing a gene’s activity below a critical threshold frequently yields insights into gene function and has been widely adopted due to the availability of Knock-out mutants via international consortia (Bradley et al. 2012) and, more recently, the use of nuclease technology has facilitated their generation (Wang et al. 2013). These strategies, however, can be challenging when exploring families of genes with similar function as compensatory effects by related family members can mask phenotypes (Barbaric et al. 2007). A gain-of-function approach frequently overcomes these redundancy problems and has proven to be an informative strategy for the analysis of gene function in many model organisms (Prelich 2012).

Despite the potential utility, gene overexpression in the mouse remains a cumbersome approach, mainly due to the methodologies used in model generation. The traditional approach, pronuclear injection, results in the random integration of the transgenic construct at varying copy number (Palmiter et al. 1986). This uncontrolled event can lead to mutagenesis (Beier et al. 1989), and frequently, transgene expression is influenced by sequences flanking the integration site (Dobie et al. 1996; Hatada et al. 1999). Multiple independent lines must be characterized to causally link phenotype with transgene expression, resulting in a high animal and financial cost. Although independent lines expressing the transgene at differing levels can allow phenotype severity to be correlated with the level of transgene expression, this fortuitous outcome is infrequently obtained.

Several ‘‘targeted transgenic’’ methodologies have been established to overcome the problems of random insertion which allow transgenes to be introduced at single copy into defined loci, permissive for transgene expression (Bronson et al. 1996; Soriano 1999). Endogenous and exogenous promoters have been used to drive transgene expression ubiquitously (Farley et al. 2000) and in a *Cre* recombinase-dependent manner (Nyabi et al. 2009), and their production has been facilitated by recombinase-mediated cassette exchange (RMCE) methodologies to increase the efficiency of site specific insertion (Hitz et al. 2007; Seibler et al. 2005). These approaches all lead to a more predictable outcome, yet only a single line of mice is generated with only one level of expression as defined by the promoter used. Exploring gene dosage with this method thus necessitates the generation of multiple lines of mice, made with different promoters, again resulting in high animal and financial cost.

**Fig. 1:**
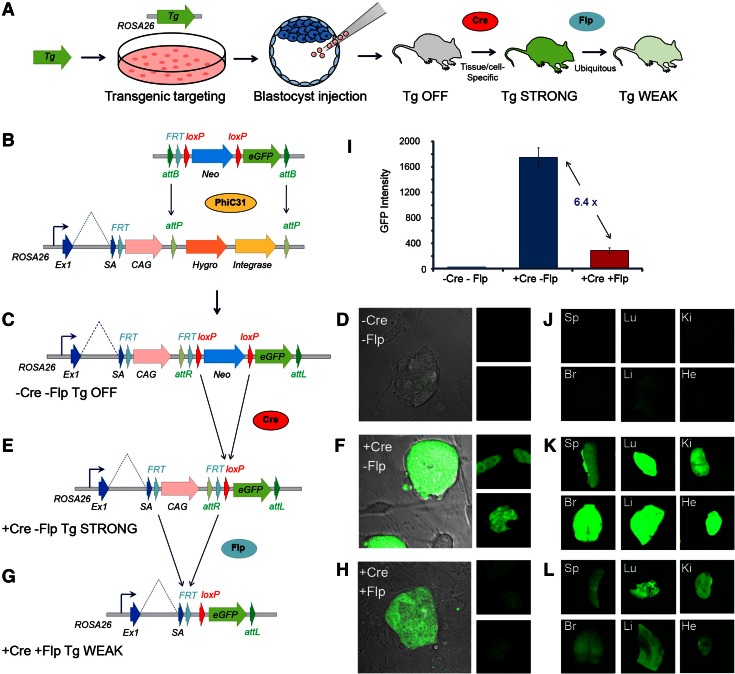
Overexpression of GFP in ES cells and mice. **a** Key steps in the generation of the promoter-switch mice. **b** *Lower panel* shows the *ROSA26* locus tagged with the PhiC31 integrase docking site. *Upper panel* shows the CB93 exchange vector with the transgene of interest positioned downstream of the loxP-flanked Neomycin cassette (Neo). **c** The *ROSA26* allele following the PhiC31 integrase-mediated cassette exchange with (**d**) ES cell GFP expression silenced. **e** The *ROSA26* allele following transgene activation by *Cre* recombinase yielding (**f**) strong ES cell GFP expression. **g** The *ROSA26* allele following the CAG promoter deletion by *Flp* recombinase, coupling transgene expression to the *ROSA26* promoter and yielding (**h**) a visibly lower level of ES cell GFP expression. **i** Quantification of ES cell GFP fluorescence before and after *Cre* and *Flp* recombinases. **j** Spleen (Sp), lung (Lu), kidney (Ki), brain (Br), liver (Li) and heart (He) GFP expression before recombinase action; **k** after *Cre* recombinase activation and (**l**) after *Flp* recombinase promoter switching

Here we report the development of an improved targeted transgenic methodology which generates a more versatile overexpression allele, which we call the promoter-switch allele, capable of driving transgene expression conditionally and at two different expression levels. The method allows the advantages of transgenic targeting approaches to be combined with an ability to explore the effects of transgene dosage, yet only a single line of mice need be generated per construct. Using our system, transgenes are efficiently targeted to the neutral *ROSA26* (*Gt*(*ROSA*)*26Sor*) locus via PhiC31 integrase RMCE (Chen et al. 2011) where their expression is initially silenced. Upon *Cre* recombinase activation, the transgene expression becomes linked to the strong CAG promoter, allowing conditional tissue-specific transgene overexpression. The action of *Flp* recombination then substitutes the strong CAG promoter (Niwa et al. 1991) for the weaker *ROSA26* promoter (Soriano 1999) allowing the effects of more moderate transgene expression to be investigated (Fig. 1a).

## Materials and methods

### Generation of the RS-PhiC ES cell line

For the promoter-switch docking site, a targeting vector previously used to equip the *ROSA26* locus for PhiC31 integrase cassette exchange, pRosa26.10 (Hitz et al. 2007), was adapted by firstly replacing the FRT-attP-PGK-Hygro-attP-3′-homology arm-dTA, released as a *Pac*I-*Kpn*I fragment with a synthesized *Asi*SI-*Kpn*I linker, FRT-MfeI-*Pac*I-*Sal*I-*Eco*RI-attP-*Xho*I-*Nsi*I. Into the MfeI-*Pac*I sites of this vector, the CAG promoter, released as an *Eco*RI-*Pac*I fragment from pCAG-C31(NLS)-bpA (Hitz et al. 2007), was cloned, generating plasmid pRosa-CAG. An assembly of Hygro-E2A-PhiC31-integrase-P2A-dsRed-SV40-pA was generated by fusion PCR using pRosa26.10 as a template for the Hygromycin selection cassette, pPGKPhiC31obpA (Addgene #13795) as a template for a murine codon-optimized PhiC31 integrase and pSIREN-DNR-dsRed-Express (Clontech) as a template for the dsRed-SV40 pA and cloned into the *Sal*I-*Eco*RI sites in pRosa-CAG. The 3′ homology arm and dTA negative selection cassette from pROSA26.10 were released as a *Sac*II-*Kpn*I fragment and inserted into the same sites in a cloning vector, pKO Scrambler 924 (Stratagene), allowing its release as an *Xho*I fragment and subsequent insertion into the *Xho*I site of the above assembly, completing the final targeting vector. The final vector included a dsRed cassette linked to the PhiC31 integrase by a P2A self-cleaving peptide. Although the CAG-Hygro-E2A-PhiC31 integrase-P2A-dsRed-pA construction yielded red fluorescence when tested in transient transfection of HEK293T cells, upon genomic integration, no dsRed fluorescence above background levels could be detected. Since this fluorescent cassette is not relevant for the method, the cassette has been omitted from the main figures for clarity. The targeting vector was linearized by *Nsi*I digestion and electroporated into 1 × 10^7^ JM8F6 cells at 500 V, 3 μF. Cells were plated on mitotically inactivated mouse embryonic fibroblasts, prepared from DR4 homozygous embryos, and recombinant clones were recovered by selection in 75 μg/ml Hygromycin. Homologously recombined clones were identified by long-range PCR at the 5′ end using a forward primer, R26-5F1, binding upstream of the homology region (5′-GGCACTACTGTGTTGGCGGA-3′) and a reverse primer, R26-5R1, binding in the inserted splice acceptor sequence (5′-GGCCAGCTTATCGATACCGT-3′) which yielded a 1.6 kb product (Supplementary Fig. 1c, d). Recombination at the 3′ end was then confirmed by long-range PCR using a forward primer, PhiC31-F1, binding within the PhiC31 integrase cassette (5′-CACCGGCCCCAAGTCCTGGT-3′) and a reverse primer, Rosa3-R1, binding downstream of the 3′ homology arm (5′-CTCAGTGGCTCAACAACACTTGGTCA-3′) which yielded a 7.2 kb product (Supplementary Fig. 1c, e). Single copy integration of the vector was confirmed by Southern blot analysis using a probe against hygromycin. The targeted *ROSA26* locus was attributed the allele name *Gt*(*ROSA*)*26Sor*^*tm*1(*CAG*-*Hygro*-*PhiC31*)*Wthg*^. The targeting vector used to generate the cassette exchange-tagged *ROSA26* locus has been deposited at Addgene (Cambridge, USA), and the RS-PhiC ES cell line is available from the authors on request.

### Construction of promoter-switch exchange vectors

A 160 bp *Xma*I fragment, encoding a 3′ portion of attB-FRT-loxP-*Pst*I-*Nsi*I-loxP-*Xho*I, was synthesized and cloned between the *Xma*I sites in pCB92 (Chen et al. 2011). This reconstituted the 5′ attB site and allowed the ORF of the neomycin phosphotransferase gene together with the bovine growth hormone polyadenylation sequence (obtained from pCB92) to be inserted between the loxP sites in the resulting vector via the *Pst*I-*Nsi*I sites. A polylinker of unique restriction sites, allowing easy cloning of transgenes, was then added together the 3′ attB site as a synthesized linker via the *Xho*I site, creating the promoter-switch exchange vector pCB93.

To generate the GFP exchange construct, an eGFP-rabbit-beta-globin-pA sequence was obtained from pCx-eGFP as an *Eco*RI-*Hind*III fragment and cloned into the polylinker of pCB93, creating plasmid pCB93-eGFP. For the *Nonagouti* exchange construct, the mouse open reading frame was amplified from image clone 3154863 (Source Bioscience) using primers NA-F1 (5′-CGAATTCGTGCTGCTTCTCAGGATGGATG-3′) and NA-R1 (5′- AAGAATTCTCAGCAGTTGGGGTTGAGTACTC-3′), which added *Eco*RI overhangs and was then used to replace the eGFP sequence in pCB93-eGFP via the *Eco*RI sites, creating plasmid pCB93-NA. The two exchange vectors, CB93-eGFP and CB93-NA have been deposited with Addgene (Cambridge, USA)

### PhiC31 integrase-mediated cassette exchange

1 × 10^6^ RS-PhiC ES cells were electroporated with 5 µg of exchange plasmid using the Neon transfection system (Life Technologies) (3 × 1400 V, 10 ms) and plated on mitotically inactivated mouse embryonic fibroblasts, prepared from DR4 homozygous embryos. After approximately 7 days of selection in 210 µg/ml G418, 24 resistant colonies were isolated per construct, expanded and screened for the correct cassette exchange event at the 5′ end, using a forward primer, CAG-F (5′-CAGCCATTGCCTTTTATGGT-3, binding within the CAG promoter, upstream of the 5′ att site) and a reverse primer, ExNeo2 (5′-GTTGTGCCCAGTCATAGCCGAATAG-3′), binding within the exchange vector in the neomycin selection cassette, to yield a 589 bp product, and at the 3′ end, using a forward primer, att-F1 (5′-GCACTAGTTCTAGAGCGATCCCC-3′), binding at the very 3′ end of the exchange vector and a reverse primer, 3HR-R1 (5′-CGGGAGAAATGGATATGAAGTACTGGGC-3′), binding downstream of the 3′ att site, to yield a 511 bp product (Supplementary Fig. 2c).

### *Cre* and *Flp* transient transfections

1 × 10^6^ ES cells harbouring the GFP promoter-switch allele were transiently transfected with either 5 µg of p*Cre*Pac (Taniguchi et al. 1998) or p*Flp*O (Addgene #13793) using the Neon transfection system (Life Technologies) (3 × 1400 V, 10 ms) and plated at low density on mitotically inactivated mouse embryonic fibroblasts. Individual colonies were isolated and screened for the deletion event. Recombined GFP clones were identified by PCR using a reverse primer, GFP-NR3, binding within the eGFP ORF (5′-ACACGCTGAACTTGTGGCCG-3′) and either the CAG-F primer to yield a 622 bp product in *Cre* recombinase-deleted clones (Supplementary Fig. 2d) or a forward primer, del-ROSA-F1 (5′-CAGCCATTGCCTTTTATGGT-3′), binding upstream of the splice acceptor to yield a 652 bp product in *Flp* recombinase-deleted clones (Supplementary Fig. 2e).

### Quantification of ES cell eGFP fluorescence

Cells were observed using a Zeiss confocal laser microscope (LSM, Carl Zeiss Microscopy) with a 63 × lens. During imaging of different groups of cells, the same system configuration was used, keeping all parameters fixed, including laser power, laser line, dichroic beam splitter for separating excitation and emission, pinhole size and scanning speed. GFP expression was quantified using the MetaMorph software (Molecular Devices). The average signal intensity of each cell was measured by tracing around individual cells. Average intensity over all of the cells was computed as the mean of all signal intensity values from each cell.

### Animals

Mice were housed in individually ventilated cages and received food and water ad libitum. All studies received local ethical review approval and were performed in accordance with UK Home Office Animals (Scientific Procedures) Act 1986. *Cre*-deleter mice (Tg(Pgk*1*-cre)1Lni (Lallemand et al. 1998)), inducible *Cre*-deleter mice (*Gt*(*ROSA*)*26Sor*^tm2(cre/ERT2)Brn^ (Hameyer et al. 2007)), *Flp*-deleter mice (Tg(*ACTB*-*Flpe*)9205Dym (Jax stock 005703)), and KRT14-*Cre* mice (Tg(*KRT14*-cre)1Amc/J (Jax stock 004782) were all maintained on a C57BL/6J background. For comparisons of weight gain, sample sizes of 7 male and 9 female mice for strong overexpression (+*Cre* −*Flp*), 8 male and 6 female mice for weaker overexpression (+*Cre* +*Flp*) and 9 male and 16 female non-expressing control mice (−*Cre* −*Flp*) were used. Fat content was measured with an EchoMRI-100 Small Animal Body Composition Analyzer, and GFP expression from whole organs was monitored using ChemiDoc™ XRS + System (Biorad). Statistically significant differences between experimental groups at fixed age points were tested with the Student’s *t* test. *Cre*-ERT2 activation was induced by 5 daily 100 µl i.p. injections of 4-hydroxytamoxifen dissolved in corn oil (Sigma) at a dose of 75 mg/kg body weight.

### Mouse production and deleter matings

A single line of ES cells harbouring the GFP promoter switch (E04) and a single line of ES cells harbouring the *Nonagouti* promoter-switch allele (B04) were microinjected into albino C57BL/6J (Jax stock 000058) blastocysts, and a chimaera showing >90 % ES cell contribution was obtained in both cases. The chimaeras were mated with albino C57BL/6J females, and germline transmission was confirmed in the F1 generation by the presence of black pups. These were genotyped with the primer combination used for PhiC31 integrase-mediated cassette exchange. An F1 heterozygote harbouring the promoter-switch allele was obtained in the 1st litter (GFP) or the 2nd litter (*Nonagouti*) following test crossing of these chimaeras. These F1 mice were then bred with *Cre*-deleter mice, and successful deletion of the loxP-flanked neomycin selection cassette was detected by PCR as described above for eGFP promoter-switch allele. For the *Nonagouti* promoter-switch allele, *Cre*-deleted clones were identified using the CAG-F primer in combination with the NA-R1 primer, yielding a 944 bp product (Supplementary Fig. 3b), and *Flp*-deleted clones were identified using the del-Rosa-F1 primer in combination with the NA-R1 primer, yielding a 974 bp product (Supplementary Fig. 3c).

To remove the recombinase transgenes and avoid the complication of mosaicism resulting from incomplete deletion events, the *Cre-* and *Flp*-deleted lines were backcrossed with C57BL/6J prior to analysis and targeted transgenic mice which had not inherited the recombinase transgenes were taken for further analysis. Southern blot analysis was performed on genomic DNA from mice harbouring the *Cre*-deleted allele and the *Flp*-deleted allele which validated the recombination events and excluded the possibility of multiple integration of the original exchange plasmid in the lines (data not shown).

### Expression analysis

Total RNA was isolated from homogenized tissue (Qiashredder, Qiagen) using the RNeasy Mini Kit (Qiagen), and first-strand cDNA was synthesized using Tetro reverse transcriptase (Bioline) as per manufacturer’s instructions using 500 ng of total RNA. Q-PCR analysis of *Nonagouti* transcripts was performed using the primer pair (5′-CTTCCAAGAAAAAGGCTTCG-3′ and 5′- ATTCTCAGCAGTTGGGGTTG-3) with normalization against two housekeeping genes: *Hprt* (5′-AGCTACTGTAATGATCAGTCAACG-3′ and 5′-AGAGGTCCTTTTCACCAGCA-3′) and *Gapdh* (5′-TGCGACTTCAACAGCAACTC-3′ and 5′-CTTGCTCAGTGTCCTTGCTG-3′) using the Power SYBR Green PCR Master mix (Applied Biosystems) and a BioRad CFX96 cycler as per manufacturer’s instructions. Relative expression was calculated using the Livak method.

Tissues were pulverized on dry ice and lysed in 50 mM Tris, 150 mM NaCl, 0.5 % Triton X100 containing protease inhibitors (complete, EDTA-free Protease Inhibitor Cocktail, Roche) at 4 °C for 20 min, followed by centrifugation and removal of the non-soluble pellet. Protein concentrations were quantified using the Bicinchoninic acid (BCA) protein assay kit (Pierce). 30 µg of whole cell lysate was loaded into each well of an Amersham ECL 8–16 % Polyacrylamide gel (GE Healthcare) and blotted onto a nitrocellulose membrane using standard protocols. Mouse monoclonal antibody against eGFP (Roche #11814460001) and mouse monoclonal antibody against *α*-tubulin (Tat-1; Sigma #00020911) were used for protein detection.

## Results

### Preparation of the docking site cell line and the RMCE exchange vector

A C57BL/6 ES cell line, RS-PhiC, harbouring a docking site for PhiC31 integrase-mediated cassette exchange at the *ROSA26* locus was prepared by gene targeting (Fig. 1b and Fig. S1). A CAG promoter-driven hygromycin resistance and PhiC31 integrase construct were targeted to intron 1 of the *ROSA26* locus. PhiC31 attP sites were positioned around the Hygro-E2A-PhiC31 coding region, constituting the docking site for the subsequent cassette exchange. A splice acceptor and an FRT site were included upstream of the CAG promoter to allow transgenes to be coupled to the endogenous *ROSA26* promoter (see below).

An exchange vector, CB93, harbouring a loxP-flanked promoterless neomycin resistance cassette upstream of a polylinker into which transgenes can be cloned, flanked by PhiC31 attB sites, was prepared (Fig. 1b). An additional FRT site was included upstream of the floxed neomycin cassette which provides the basis of the promoter switching in subsequent steps.

### In vitro proof-of-concept using an eGFP reporter in ES cells

As a first test, GFP was cloned into CB93, and the resulting exchange vector was electroporated into RS-PhiC ES cells. PhiC31 integrase expressed from within the docking site catalysed the recombination between the genomic attP sites and the exchange vector attB sites, leading to an exchange of the Hygro-E2A-PhiC31 integrase cassette for the FRT-loxP-Neo-loxP-eGFP cassette (Fig. 1b and Fig. S2a–c), conveying G418 resistance. The presence of the PhiC31 expression cassette obviates the need to introduce exogenous integrase to mediate exchange and the exchange event leads to the excision of the cassette itself. Consequently, the efficiency of the cassette exchange event is very high and, routinely following electroporation of 1 × 10^6^ RS-PhiC ES cells, several hundred G418 resistant colonies were obtained. Similar to our previously reported results with a PhiC31 cassette exchange system at *ROSA26* (Chen et al. 2011), all G418 resistant clones represent a targeted integration event, with approximately 70 % of the colonies harbouring a true exchange event (5′ and 3′ attP × attB recombination occurring), the remaining 30 % had integrated the entire vector via a single 5′ attP × attB recombination event. The system thus allows a targeted integration efficiency of at least 1 × 10^−4^ with respect to the cell population electroporated.

Correct exchange led to the GFP cassette being positioned downstream of the CAG promoter (previously used to drive the Hygro-E2A-PhiC31 integrase expression) (Fig. 1c). Expression of eGFP was initially hindered by the presence of the loxP-flanked neomycin cassette, the polyadenylation sequence of which blocks transcription (Fig. 1d). To test the *Cre*-dependent activation of transgene expression, cassette-exchanged clones were transiently transfected with *Cre* recombinase. Strong GFP expression resulted in *Cre*-deleted clones due to the excision of the neomycin cassette, linking the strong CAG promoter to GFP (Fig. 1e, f). Next, the *Flp* recombinase promoter switch was tested by transiently transfecting the *Cre*-deleted clones with *Flp* recombinase. *Flp* recombinase excised the FRT-flanked CAG promoter, linking the GFP to the endogenous *ROSA26* promoter via the splice acceptor lying within intron 1 (Fig. 1g). GFP expression in the resulting *Flp*-deleted clones was visibly weaker (Fig. 1h), and quantification of the fluorescence change revealed a level approximately 6.4-fold lower than in non-*Flp*-recombined clones (Fig. 1i).

### In vivo proof-of-concept using an eGFP reporter promoter in mice

**Fig. 2:**
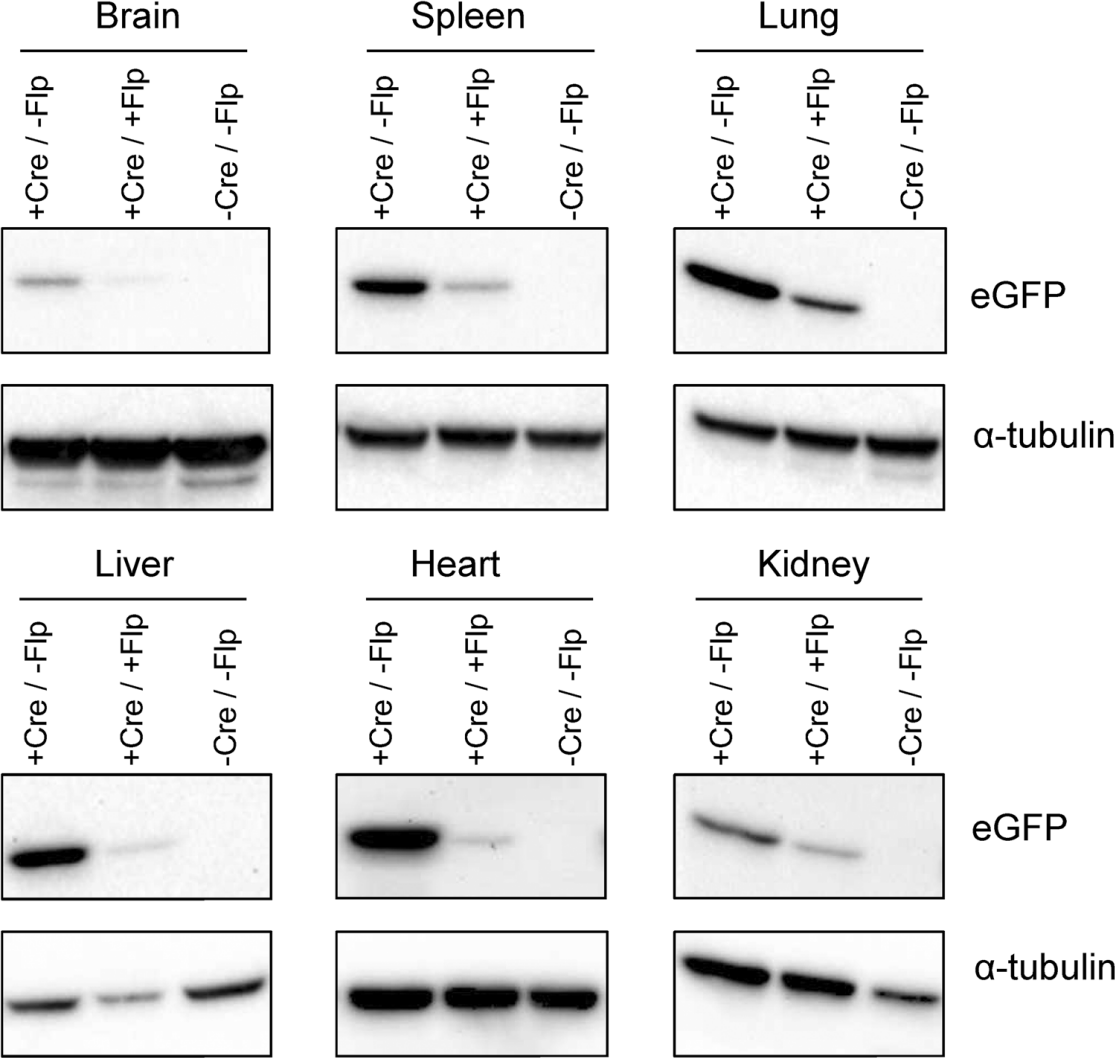
Western blot analysis of brain, spleen, lung, liver, heart and kidney of mice harbouring the eGFP overexpression allele before and after the activation by *Cre* recombinase and after the promoter switching by *Flp* recombinase. eGFP and *α*-tubulin were detected with molecular weights of approximately 28 and 55 kDa, respectively

Having demonstrated that the *Cre* recombinase activation and *Flp* recombinase promoter switching leads to robust transgene expression at two different expression levels in ES cells, the system was then tested in vivo. A mouse line harbouring the GFP promoter-switch allele was prepared by blastocyst injection of cassette-exchanged ES cells and breeding of the resulting chimaeras. As expected, these mice were not found to express GFP, due to the presence of the loxP-flanked neomycin cassette (Fig. 1j). On breeding with *Cre*-deleter mice (Fig. S2d), deleted offspring strongly expressed GFP under the control of the CAG promoter (Fig. 1k). These mice were then bred with *Flp*-deleter mice (Fig. S2e), and *Flp*-deleted offspring expressed GFP at a considerably lower level (Fig. 1l). GFP expression was analysed across several organs by Western blot analysis which confirmed the visible difference in expression level before and after *Cre* and *Flp* recombinase switching (Fig. 2), with CAG-driven expression being considerably stronger than the *ROSA26* driven expression in all tissues studied.

### Correlation of a visible phenotype and expression level in a *Nonagouti* promoter-switch model

To establish whether this method can be used to help functional analysis by allowing the severity of a phenotype to be correlated with transgene expression, mice harbouring the cDNA of the *Nonagouti* peptide in the promoter-switch allele were generated (Fig. S3). The peptide antagonizes the binding of alpha-melanocyte stimulating hormone to the melanocortin-1 receptor, switching melanin synthesis in the hair follicle from the black eumelanin to the yellow pheomelanin pigment (Bultman et al. 1992). Overexpression thus yields a discernible coat colour phenotype, as exemplified by several yellow mouse strains whose coat colour is due to *Nonagouti* gain-of-function (Miller et al. 1993).

**Fig. 3:**
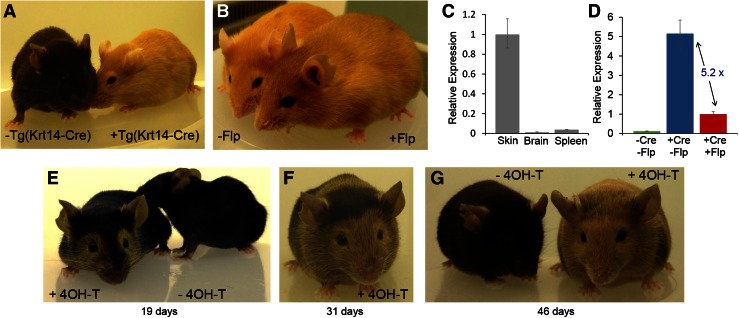
Testing the promoter-switch allele with a visible phenotype. **a** Mice harbouring the *Nonagouti* overexpression allele, with (*right*) and without (*left*) the Tg(KRT14-*Cre*) transgene. **b** Mice harbouring the *Cre*-activated *Nonagouti* overexpression allele, before (*left*) and after (*right*) the action of *Flp* recombinase. **c** Quantification of transgenic *Nonagouti* transcripts in the skin, brain and spleen of Krt14-*Cre*-activated *Nonagouti* overexpressing mice. **d** Quantification of transgenic *Nonagouti* transcripts in the skin before and after the action of *Cre* and *Flp* recombinase. **e**, **f**, **g** Mice harbouring the *Nonagouti* overexpression allele with a tamoxifen-inducible *Cre*-ERT2 transgene at 19, 31 and 48 days following the injection of 4-hydroxytamoxifen (+4-OH-T) or vehicle (-4-OH-T)

Before *Cre* recombinase treatment, mice harbouring the non-activated promoter-switch allele were black in coat colour and indistinguishable from wild-type litter mates. When these mice were crossed with Tg(KRT14-*Cre*), mice which express *Cre* recombinase in melanocytes, offspring showing a characteristic strong agouti colouring were obtained (Fig. 3a). When these mice were crossed with *Flp*-deleter mice, a more subtle agouti colour resulted (Fig. 3b). Transgene expression analysis in KRT14-*Cre*-activated mice confirmed transgenic *Nonagouti* expression in the skin, where the KRT14-*Cre* transgene is active, but, not in other tissues, where the KRT14-*Cre* transgene is not expressed, such as brain and spleen (Fig. 3c). Furthermore, differences in *Nonagouti* expression levels within the skin before and after *Flp* recombinase-mediated promoter switching, were consistent with expression driven by the strong CAG promoter (before *Flp* recombination) or the weaker *ROSA26* promoter (after *Flp* recombination) and the visible differences in coat colour before and after *Flp* expression correlated with these observed levels of skin expression (Fig. 3d). These results visibly demonstrate the flexibility of the promoter-switch methodology for achieving tissue-specific transgene expression at two different dosages.

To explore the versatility of the system in temporal control of transgene expression, a ubiquitous tamoxifen-inducible *Cre* recombinase transgene was introduced by breeding the non-recombined *Nonagouti* mice with *Gt*(*ROSA*)*26Sor*^*tm2*(*Cre*-*ERT2*)*Brn*^ mice. The mice harbouring both transgenes were black in coat colour and indistinguishable from wild-type litter mates. Double-transgenic mice were injected with 4-hydroxytamoxifen and after 2 weeks, inducible activation of strong *Nonagouti* overexpression became evident from the growth of yellow hair. Yellow hair growth progressed for the next 3–4 weeks until the mice became almost entirely yellow in appearance (Fig. 3e, f, g). The temporal activation of expression using this simple coat colour marker highlights the strict non-leaky control of transgene expression which is achievable with the promoter-switch method.

### Correlation of a quantifiable phenotype and expression level in a *Nonagouti* promoter-switch model

**Fig. 4:**
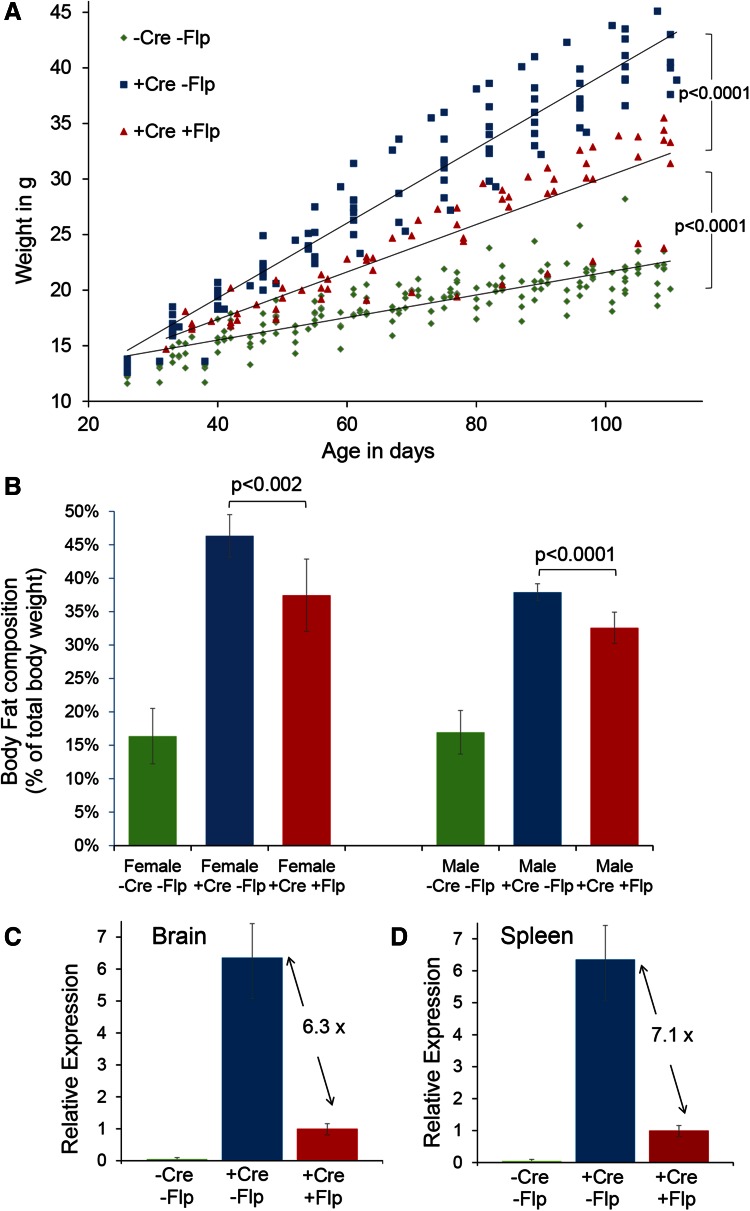
Correlating gene overexpression with phenotype severity. **a** Growth curves for female *Nonagouti* overexpression mice before (*green diamonds*; *n* = 16) and after (*blue squares*; *n* = 9) ubiquitous *Cre* recombinase activation and following the *Flp* recombinase promoter switch (*red triangles*; *n* = 6). **b** Body fat composition analysis of female and male *Nonagouti* overexpression mice before and after ubiquitous *Cre* recombinase activation and following the *Flp* recombinase promoter switch. **c**, **d** Quantification of transgenic *Nonagouti* transcripts in the brain and spleen of mice before and after ubiquitous *Cre* recombinase activation and following the *Flp* recombinase promoter switch. *p* values were calculated with the Student’s *t* test using weights and body fat composition at 110 days of age

In the above example, the different intensities of coat colour demonstrate the consequences of different gene dosages of *Nonagouti* overexpression, but we were keen to explore a more quantifiable phenotype to fully validate the technique for correlating phenotype and expression. *Nonagouti* is also known to antagonize members of the melanocortin receptor family outside of the skin follicle and within the hypothalamus, ectopic *Nonagouti* overexpression results in obesity (Klebig et al. 1995). To test the promoter-switch allele with this quantifiable phenotype, a ubiquitous *Cre*-deleter mouse was used to activate transgene expression in all tissues. These mice were then bred with *Flp*-deleter mice to switch the promoter. It quickly became apparent that *Nonagouti*-expressing mice at both expression levels were prone to obesity, but significantly the weight gain occurred at a faster rate when the transgene expression was under the control of the stronger CAG promoter, as compared to the weaker *ROSA26* promoter (Fig. 4a and Fig. S4). These mice were analysed for body fat content at approximately 16 weeks of age, and a significant association between the strength of the promoter used and body fat was found (Fig. 4b). Transgene expression, now ubiquitously activated, was quantified in two example tissues, brain and spleen and, as expected, *Flp*-deleted mice were found to express *Nonagouti* approximately sixfold and sevenfold lower than the non-*Flp*-deleted mice (Fig. 4c, d). Thus, the degree of weight gain and fat deposition, i.e. the severity of the phenotype was found to correlate with the two levels of transgene expression achieved with the promoter-switch methodology.

## Discussion

It is clear from numerous human pathologies that abnormal gene activation or copy number (Girirajan et al. 2011), which frequently leads to increased gene expression, underlies human disease states. Indeed the causative SNPs emerging from genome-wide association studies frequently map to regulatory sequences (Maurano et al. 2012; Schaub et al. 2012) and are thus thought to exert their effects by altering gene expression. How these expression changes contribute to altered cell physiology leading to disease progression remains largely unexplored. Our robust promoter-switch methodology focussed on exploring gene overexpression at different gene dosages is thus of considerable application in dissecting the molecular mechanisms underlying these potentially pathogenic increases in gene expression.

Current methods for gene overexpression in the mouse are rather blunt instruments as they provide only a single-expression level per line. Traditionally, random integration approaches have been adopted with simple promoter cDNA or Bacterial Artificial Chromosome-based constructs which lead to multiple lines of mice, each with a unique copy number and site of integration. This method is sometimes able to yield different lines with different levels of expression with which the consequences of gene dosage can be explored; however, this fortuitous outcome is infrequently obtained as high copy number transgene arrays are frequently associated with variegation and silencing (Garrick et al. 1998). Transgenic targeting—the targeting of overexpression constructs to defined permissive loci—overcomes a lot of these problems but to obtain different expression levels, multiple lines with different promoters much still be generated. Irrespective of the methodology used for their generation, while being highly informative, the study of multiple lines necessitates the generation, breeding and characterisation of several mouse models which come at a significant animal and frequently prohibitive financial cost.

Our promoter-switch methodology combines the advantages of transgenic targeting with two recombinase switches allowing a flexibility to change overexpression dosage thus providing versatile overexpression models with only a single line of mice required per gene. The first switch is a *Cre* recombinase activation which can be used to turn on strong transgene overexpression in specific target tissues and cell types, as defined by the expression pattern of the *Cre* driver, or activation can occur at a specific time point through the use of inducible *Cre* recombinase lines. *Cre* recombinase expressing lines are freely available within the scientific community (Nagy et al. 2009), and international consortia have been funded to streamline their production (Chandras et al. 2012). The second switch is provided by the action of a binary (on/off) *Flp* recombinase to change overexpression dosage and is achieved by simple breeding with a ubiquitously expressed *Flp* recombinase-deleter line, many of which are widely available (Birling et al. 2012; Rodriguez et al. 2000). For clarity, the promoter-switch method does not envisage the use of tissue-specific *Flp* recombinase expressing mice, which are rarely available, only a *Flp*-deleter mouse needs to be used for the promoter switch as the tissue specificity is provided by the *Cre* recombinase activation step.

The two levels of overexpression achieved by this methodology reflect the activities of a single copy of a CAG promoter- or a *ROSA26* promoter-driven expression construct. Although these promoters are considered to direct broadly ubiquitous expression, tissue-specific differences in expression level have been observed for reporters driven by the CAG (Kawamoto et al. 2000) and the *ROSA26* (Kisseberth et al. 1999) promoters. Consistently, in our promoter-switch models, we observed tissue-specific differences in the relative expression levels, as exemplified by the variation in eGFP expression between tissues and by the varying ratios of CAG versus *ROSA26* promoter-driven transcript levels quantified in ES cells, skin, brain and spleen, before and after *Flp* recombinase promoter switching. Although it is important to consider these tissue-specific differences in relative expression levels before and after promoter switching, it is clear that CAG-driven expression is considerably stronger than the *ROSA26*-driven expression in all tissues.

The two different expression levels achievable with the promoter-switch methodology provide considerable flexibility when studying the effects of gene overexpression. Where a phenotype is only apparent after a certain threshold of overexpression is exceeded, the promoter-switch approach allows the consequences of very strong overexpression to be studied by simple *Cre* activation (without the *Flp* recombinase promoter switch). Conversely, for example, where genes are associated with an adverse phenotype or where the endogenous expression level of a gene is low, it might be more appropriate and biologically relevant to consider a milder level of overexpression. With the promoter-switch methodology, performing the *Flp* recombinase promoter switch in advance of tissue-specific *Cre* activation allows a weaker level of overexpression to be achieved. Phenotype and expression level are rarely linearly correlated, and subsequently our promoter-switch method which achieves two extremes of gene overexpression in a single mouse model provides considerable utility for probing gene function.

The cassette exchange approach at the heart of the promoter-switch method would be amenable to high-throughput applications and systematic screening studies. The constitutive expression of the PhiC31 integrase enzyme from within the cell line, driving the transgene integration event and the concomitant deletion of the integrase in correctly targeted clones, streamlines the whole production process and greatly increases the efficiency of targeted insertion by avoiding the necessity to introduce exogenous integrase. With our system, we report targeted integration efficiencies of at least 1 × 10^−4^ with respect to the starting ES cell population electroporated, 2–3 orders of magnitude above the rates recorded for conventional gene targeting.

With respect to conversion of the ES cell resource into mouse lines, with the two lines prepared in this study, chimaeras with over 90 % ES cell contribution (by coat colour) were obtained for both projects following a single injection session and transfer of a single foster mother with microinjected embryos. Germline transmission was obtained for both ES cell lines by breeding just a single chimaera. The results we obtained in this study compare favourably with our experience of targeted ES cell clones generated on the JM8F6 background, for example, those obtained from the International Knock-out Mouse Consortium (IKMC) resource, and are consistent with the production efficiencies reported by other laboratories working with this parental line (Pettitt et al. 2009). Laboratories having gained experience with the IKMC resource would therefore be expected to be able to work with cassette-exchanged RS-PhiC ES cells using the standard conditions recommended for JM8F6 ES cell injection (https://www.komp.org/protocols.php). For an even faster production, previous studies have used a PhiC31 integrase RMCE approach directly in the oocyte, through the co-microinjection of the exchange vector together with PhiC31 integrase mRNA in zygotes genetically harbouring the docking site (Ohtsuka et al. 2010). Our promoter-switch allele could be generated in this fashion, and future studies will address the feasibility of implementing this refined technique, obviating the need for ES cell work.

Mouse breeding can be a potential bottle neck in functional analysis and the allelic conversion, i.e. activation of the transgene by *Cre* recombinase and, when required, the switching of promoter strength by *Flp* recombinase would indeed require further mouse generations. However, since only a single (heterozygous) copy of the promoter-switch allele is required for strong overexpression, the first experimental cohorts of mice with *Cre*-activated strong overexpression, together with their necessary controls, would be available within a single generation (3–4 months). Where ubiquitous activation is appropriate, techniques exploring the use of cell-permeable recombinases for allelic conversion, as reported for the Knock-out first alleles (Ryder et al. 2014) could be applied to speed up the availability of cohorts.

In summary, the promoter-switch approach has proven to be a robust, reliable and versatile method of studying gene overexpression. Firstly, the PhiC31 integrase-mediated cassette exchange approach provides an efficient means of generating targeted transgenic lines and is particularly well suited for the comparison of multiple lines of mice expressing variant transgenes. Secondly, spatiotemporal transgene overexpression can be achieved through the use of tissue-specific and inducible *Cre* recombinase expressing mice. Thirdly, through the use of *Flp*-deleter mice, two lines of mice can be generated allowing strong overexpression, driven by the CAG promoter, to be compared with more moderate expression levels, driven by the endogenous *ROSA26* promoter. Thus, a single line of mice needs only to be generated per transgene, and through a series of simple crosses with recombinase expressing lines, informative overexpression models can be generated, allowing phenotypic severity to be correlated with gene dosage.

## Electronic supplementary material

Supplementary material 1 (PDF 1947 kb)
