# Supplementary material for: A versatile transgenic allele for mouse overexpression studies
Source: Mamm Genome. 2015 Sep 14;26(11-12):598–608. doi: 10.1007/s00335-015-9602-y (PMC4653235; doi:10.1007/s00335-015-9602-y)
Supplement: Supplementary file 1 — Supplementary material 1 (PDF 1947 kb) [file 335_2015_9602_MOESM1_ESM.pdf]

Figure S1

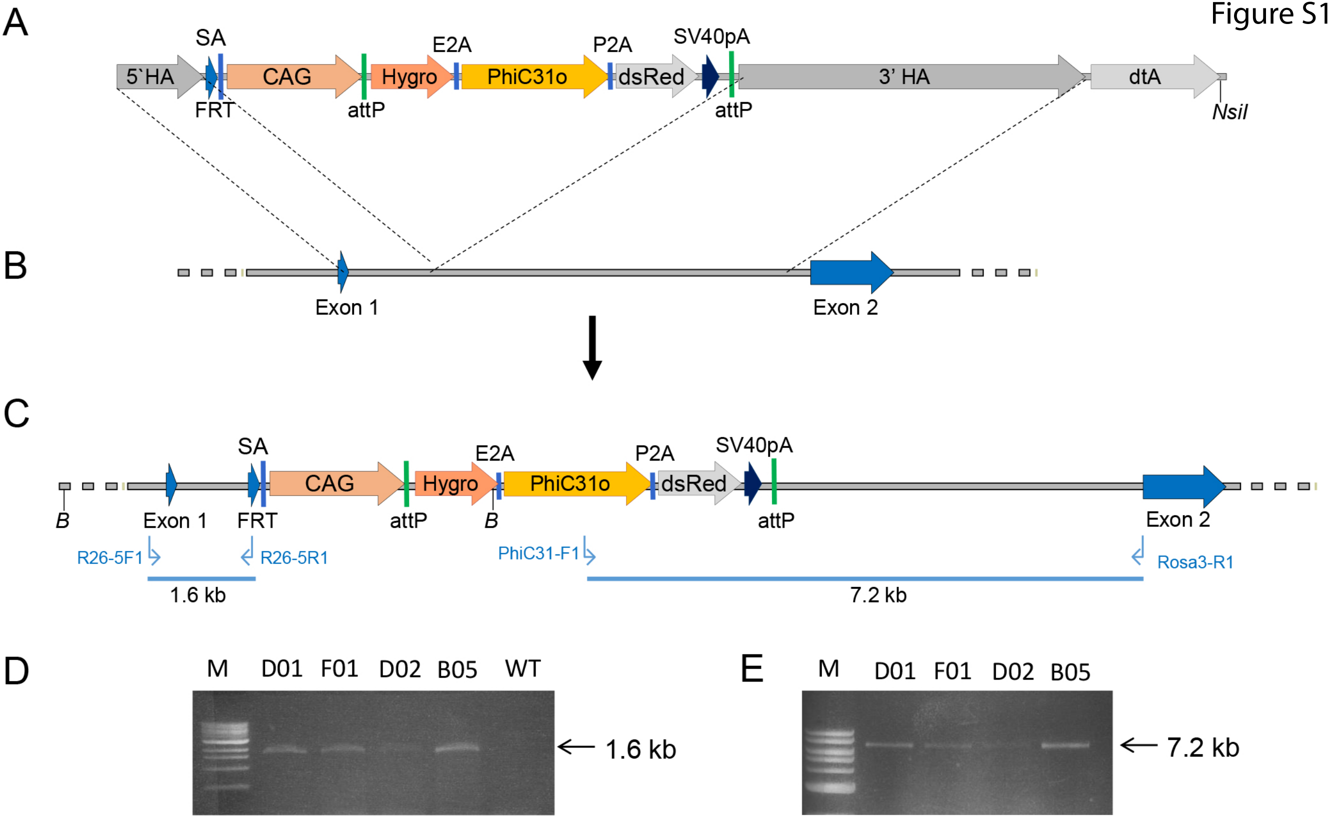

A
SA
E2A
P2A
SV40pA
5`HA
CAG
Hygro
PhiC31o
dsRed
3' HA
dtA
FRT
attP
attP
NsiI
B
Exon 1
Exon 2
C
SA
E2A
P2A
SV40pA
CAG
Hygro
PhiC31o
dsRed
B
Exon 1
FRT
attP
B
attP
Exon 2
R26-5F1
R26-5R1
PhiC31-F1
Rosa3-R1
1.6 kb
7.2 kb
D
M
D01
F01
D02
B05
WT
1.6 kb
E
M
D01
F01
D02
B05
7.2 kb

Figure S2

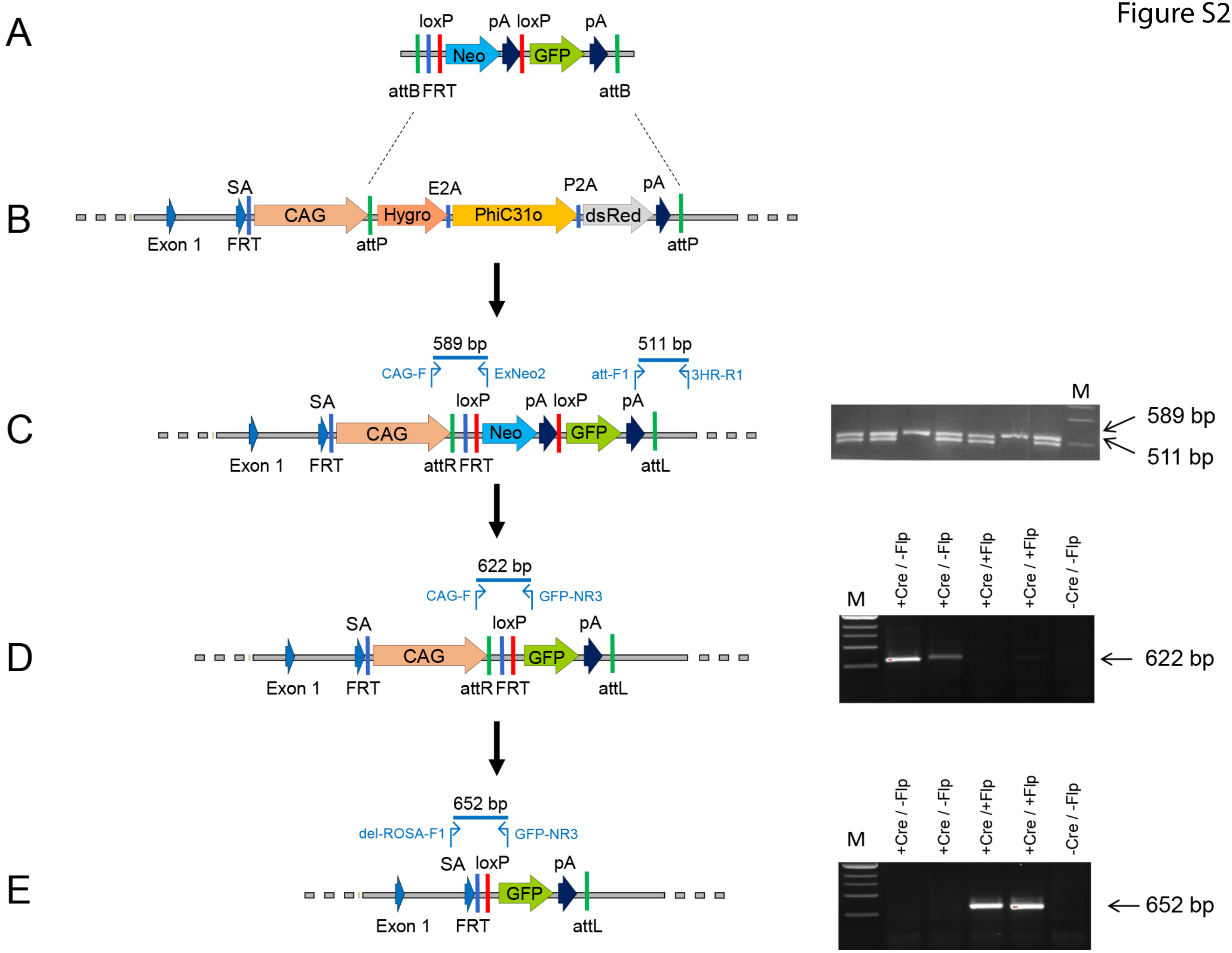

A
loxP
pA
loxP
pA
Neo
GFP
attB
FRT
attB
B
SA
E2A
P2A
pA
CAG
Hygro
PhiC31o
dsRed
Exon 1
FRT
attP
attP
C
589 bp
511 bp
CAG-F
ExNeo2
att-F1
3HR-R1
SA
loxP
pA
loxP
pA
CAG
Neo
GFP
Exon 1
FRT
attR
FRT
attL
M
589 bp
511 bp
D
622 bp
CAG-F
GFP-NR3
SA
loxP
pA
CAG
GFP
Exon 1
FRT
attR
FRT
attL
M
+Cre / -Flp
+Cre / -Flp
+Cre /+Flp
+Cre / +Flp
-Cre / -Flp
622 bp
E
652 bp
del-ROSA-F1
GFP-NR3
SA
loxP
pA
GFP
Exon 1
FRT
attL
M
+Cre / -Flp
+Cre / -Flp
+Cre /+Flp
+Cre / +Flp
-Cre / -Flp
652 bp

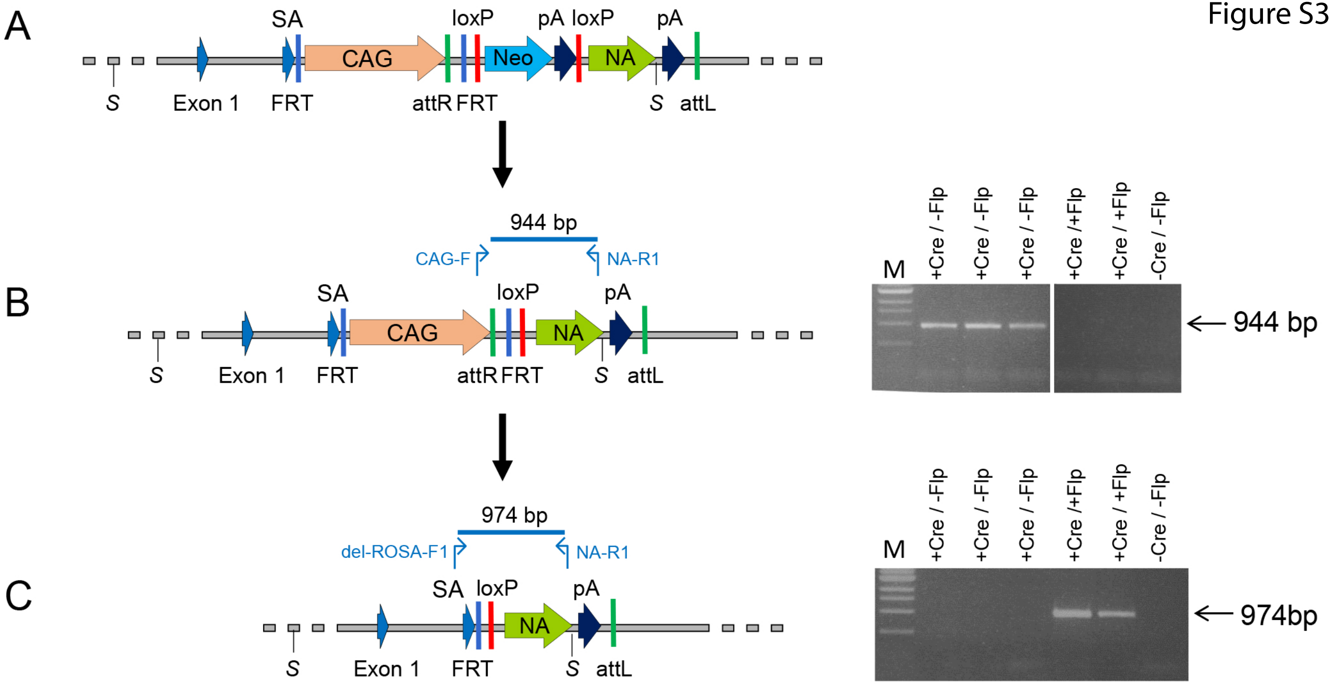
Figure S3
A
SA
loxP
pA
loxP
pA
CAG
Neo
NA
S
Exon 1
FRT
attR
FRT
S
attL
B
944 bp
CAG-F
NA-R1
SA
loxP
pA
CAG
NA
S
Exon 1
FRT
attR
FRT
S
attL
C
974 bp
del-ROSA-F1
NA-R1
SA
loxP
pA
NA
S
Exon 1
FRT
S
attL
M
+Cre / -Flp
+Cre / -Flp
+Cre / -Flp
+Cre /+Flp
+Cre / +Flp
-Cre / -Flp
944 bp
M
+Cre / -Flp
+Cre / -Flp
+Cre / -Flp
+Cre /+Flp
+Cre / +Flp
-Cre / -Flp
974bp

Figure S4

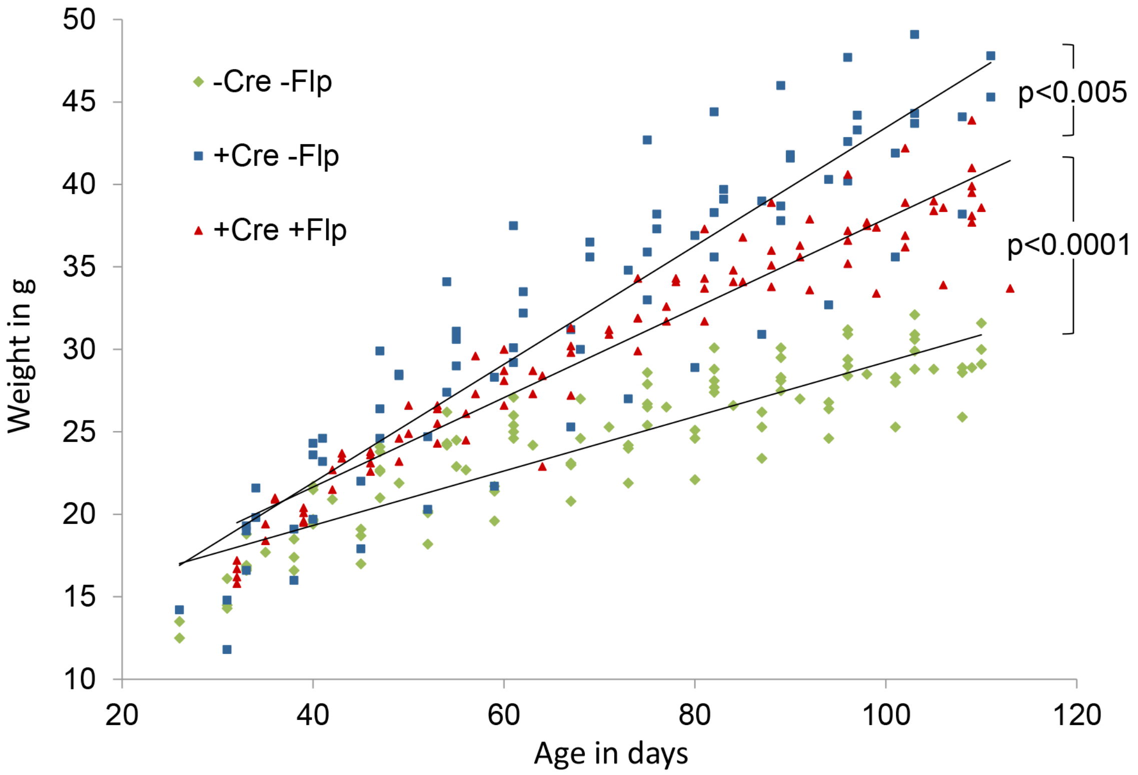

-Cre -Flp
+Cre -Flp
+Cre +Flp
p<0.005
p<0.0001
Weight in g
Age in days
50
45
40
35
30
25
20
15
10
20
40
60
80
100
120

Supplementary Figure Legends

Figure S1 Generation of the RS-PhiC ES cell line (**A**) Targeting vector used to introduce the cassette exchange machinery into the *ROSA26* locus. HA, homology arms; SA, splice acceptor; E2A and P2A self-cleaving peptides. (**B**) Endogenous *ROSA26* locus. (**C**) Targeted *ROSA26* locus showing the positions of the primers used for long-range PCR analysis to detect the targeting event. (**D**) PCR confirmation of 5' targeting using primers R26-5F1 and R26-5R1 and (**E**) PCR confirmation of 3' targeting using primers PhiC31-F1 and Rosa3-R1.

Figure S2 Generation of the GFP promoter-switch allele (**A**) Cassette exchange vector, CB93-GFP. (**B**) *ROSA26* locus targeted with the cassette exchange machinery. (**C**) Left panel shows the cassette exchanged *ROSA26* locus, showing the integration of the GFP transgene and the excision of the Hygro-PhiC31-dsRed cassette, with the primers used for PCR screening of the integration events over the 5' attR and the 3' attL sites. Right hand panel shows the results of a typical screen showing the integration of the transgene array by cassette exchange (amplicons for 5' attR and 3' attL are present) or simple integration (only amplicon for 5' attR site is present). (**D**) Cre recombinase dependent deletion of the Neo cassette activating the expression of GFP. The allele conformation with primers for the screening of the Cre deletion event are shown in the left panel and the right panel shows some example genotyping. (**E**) Flp recombinase dependent deletion of the CAG promoter, linking the expression of GFP to the endogenous *ROSA26* promoter via the splice acceptor (SA). The allele conformation with primers for the screening of the Flp deletion event are shown in the left panel and the right panel shows some example genotyping.

Figure S3 Generation of the *Nonagouti* promoter-switch allele (**A**) The cassette exchanged *ROSA26* locus, showing the integration of the *Nonagouti* (NA) transgene. (**B**) Cre recombinase dependent deletion of the Neo cassette activating the expression of *Nonagouti*. The allele conformation with primers for the screening of the Cre deletion event are shown in the left panel and the right panel shows some example genotyping. (**C**) Flp recombinase dependent deletion of the CAG promoter, linking the expression of *Nonagouti* to the endogenous *ROSA26* promoter via the splice acceptor (SA). The allele conformation with primers for the screening of the Flp deletion event are shown in the left panel and the right panel shows some example genotyping.

Figure S4 Growth curves for male *Nonagouti* overexpression mice before (green diamonds; n=9) and after (blue squares; n=7) ubiquitous Cre recombinase activation and following the Flp recombinase promoter-switch (red triangles; n=8). p values were calculated with the Student's t-test using weights at 110 days of age.
